# (A)musicality in Williams syndrome: examining relationships among auditory perception, musical skill, and emotional responsiveness to music

**DOI:** 10.3389/fpsyg.2013.00525

**Published:** 2013-08-16

**Authors:** Miriam D. Lense, Carolyn M. Shivers, Elisabeth M. Dykens

**Affiliations:** ^1^Vanderbilt Kennedy Center, Vanderbilt UniversityNashville TN, USA; ^2^Psychology and Human Development, Vanderbilt UniversityNashville, TN, USA

**Keywords:** Williams syndrome, music, amusia, pitch perception, auditory sensitivity

## Abstract

Williams syndrome (WS), a genetic, neurodevelopmental disorder, is of keen interest to music cognition researchers because of its characteristic auditory sensitivities and emotional responsiveness to music. However, actual musical perception and production abilities are more variable. We examined musicality in WS through the lens of amusia and explored how their musical perception abilities related to their auditory sensitivities, musical production skills, and emotional responsiveness to music. In our sample of 73 adolescents and adults with WS, 11% met criteria for amusia, which is higher than the 4% prevalence rate reported in the typically developing (TD) population. Amusia was not related to auditory sensitivities but was related to musical training. Performance on the amusia measure strongly predicted musical skill but not emotional responsiveness to music, which was better predicted by general auditory sensitivities. This study represents the first time amusia has been examined in a population with a known neurodevelopmental genetic disorder with a range of cognitive abilities. Results have implications for the relationships across different levels of auditory processing, musical skill development, and emotional responsiveness to music, as well as the understanding of gene-brain-behavior relationships in individuals with WS and TD individuals with and without amusia.

## INTRODUCTION

Williams syndrome (WS) is a neurodevelopmental disorder caused by the deletion of 26–28 genes on chromosome seven and has an estimated prevalence of one in 7,500 births (Strømme et al., 2002). Research into WS has become increasingly popular because its known genetic etiology and unique cognitive-behavioral profile allows for study of gene-brain-behavior links. Though cognitive abilities range from moderate intellectual disability to average, WS is usually associated with mild to moderate cognitive impairment with typical IQ in the 50s–60s (e.g., Bellugi et al., 2000; see Martens et al., 2008 for a review). IQ appears to be relatively stable with age (Howlin et al., 1998; Porter and Dodd, 2011; Mervis et al., 2012) with greater verbal than spatial abilities (Bellugi et al., 1994). Receptive language abilities appear to be a relative strength in WS while other aspects of language are consistent with their cognitive profile (Brock, 2007). WS is further characterized by anxiety (Dykens, 2003), attention problems (Rhodes et al., 2010), and hypersociability and empathic behavior (Zitzer-Comfort et al., 2007).

Williams syndrome has been of interest to music cognition researchers because of the auditory sensitivities prevalent in the syndrome, as well as evidence of greater interest and emotional responsiveness to music. Compared to typically developing (TD) individuals and those with other disabilities, individuals with WS show higher rates of hyperacusis (lowered hearing thresholds), odynacusis (pain in response to sounds), auditory fascinations, and auditory aversions (Levitin et al., 2005). Auditory aversions appear to be particularly common, with between 85 and 95% of individuals with WS reporting aversions to one or more sounds versus less than 3% of TD individuals (Klein et al., 1990; Van Borsel et al., 1997; Levitin et al., 2005). Heightened emotional responsiveness to music has also been noted in WS in comparison with TD individuals and those with other disability syndromes (e.g., von Arnim and Engel, 1964; Levitin et al., 2004; Dykens et al., 2005), with support from neuroimaging studies finding greater activation of emotion-related areas of the brain in response to music in WS (Levitin et al., 2003; Thornton-Wells et al., 2010).

Despite early reports of seemingly preserved musical abilities in WS (e.g., von Arnim and Engel, 1964; Lenhoff et al., 1997; Lenhoff et al., 2001), recent research has suggested a more nuanced profile of relative strengths and weaknesses in musical skills in WS. Among pitch and rhythm perception tasks, most studies have used formal musical assessment scales (e.g., Gordon Primary Measures of Music Audiation (PMMA; Gordon, 1986); Bentley Measures of Musical Abilities (Bentley, 1985)) that require participants to listen to pairs of notes or melodic sequences and respond if they are the same or different. Results from these types of tasks have generally indicated pitch and rhythm perception abilities commensurate with mental age (Don et al., 1999) but impaired abilities in comparison to chronological age-matched TD peers (Hopyan et al., 2001; Martens et al., 2010). However, these same/different tasks require working memory because participants must hold the original notes or melodies in mind to compare with the second melody in the pair. Individuals with WS have poorer auditory working memory skills than expected given their receptive language abilities (Don et al., 1999; Rhodes et al., 2011), thus limiting interpretations of these findings and their relationship with cognitive abilities.

Studies have also suggested that individuals with WS process musical information differently than TD individuals. While TD individuals in the general population are evenly split between being fundamental pitch processors (i.e., perceive the sound based on the fundamental frequency) or spectral pitch processors (i.e., perceive the sound by decomposing it into its harmonics; e.g., Schneider et al., 2005), Wengenroth et al. (2010) reported an extreme fundamental pitch processing bias in WS. Specifically, 27 of 29 individuals with WS were fundamental pitch processors, and to a more extreme extent than the TD fundamental pitch processors. Wengenroth et al. (2010) suggest that this perceptual difference in WS may be related to increased leftward asymmetry of the auditory cortex, particularly Heschl’s gyrus. Whereas TD children and adults show superior performance in judging pairs of melodies to be the same or different when contour rather than interval changes are present, individuals with WS do not show this advantage (Deruelle et al., 2005; Elsabbagh et al., 2010).

Fewer studies have examined musical production skills in WS. A first study of just eight children or adults with WS attending a music camp found they could clap back rhythmic patterns as well as TD younger children (Levitin and Bellugi, 1998), though the TD group was not formally matched to the WS group. Another study of 25 individuals with WS found they could clap in time to the beat of musical excerpts as well as chronological age-matched TD participants (Martens et al., 2010). In contrast, compared to TD controls, these same WS participants had impaired reproduction of rhythmic and melodic excerpts (Martens et al., 2010). Again, findings from musical production research seem to support a relative strength in musical abilities in WS though interpretations are hindered due to potential confounds of task design, control groups, and musical training.

Research also suggests that people with WS show marked variability in musical abilities but this variability is not well studied or understood. Lense and Dykens (2012) rated 46 individuals with WS when they performed a song of their choice when either singing or playing an instrument on which they had training. They found a wide variety of musical abilities, which were associated with musical training and time currently spent singing or playing an instrument. Additionally, the participants’ musical abilities on a song and instrument of their choice predicted their abilities to learn a novel musical instrument in one semi-structured lesson. Martinez-Castilla and Sotillo (2008) reported greater singing abilities in four individuals with WS with musical training when compared to 11 without training. On a variety of musical perception and production tasks, Martens et al. (2010) reported that only a small subgroup of individuals with WS performed commensurate with chronological age-matched peers while the remaining participants were impaired. Only one individual with WS demonstrated commensurate abilities in both perception and production tasks. Thus, more research is needed to understand the variability in musical abilities in WS and how abilities compare across the perception and production domains.

Although a lore persists about musical talent in WS, it should not be too surprising that music abilities range widely in WS. In the general population, some TD individuals demonstrate surprising amounts of musicality even without formal musical training, while other individuals appear to be less proficient. For example, when asked to sing a well-known song from memory, most occasional or non-singers do not perform as accurately as professional singers but still exhibit relative proficiency, rarely deviating by more than one semitone on pitch intervals or making more than four time errors (Dalla Bella et al., 2007). Moreover, as occasional singers tend to sing much faster than professionals, their performance accuracy improves to the level of professionals when they are forced to sing at a slower tempo (Dalla Bella et al., 2007). However, a few individuals remain markedly inaccurate, making more than 10 times the number of pitch interval errors than other occasional singers (Dalla Bella et al., 2007).

In the TD population, individuals who show marked impairment in pitch perception abilities despite otherwise intact cognitive functioning are considered to have amusia, also known as tune/tone-deafness (Drayna et al., 2001; Peretz et al., 2003). Though amusia can result from neurological damage such as a stroke (i.e., acquired amusias; e.g., Särkämö et al., 2010), amusia may also be congenital in nature. The estimated prevalence of congenital amusia in the TD population is approximately 4% (Kalmus and Fry, 1980; Sloboda et al., 2005), and amusia appears to have genetic associations (Drayna et al., 2001; Peretz et al., 2007). Amusic individuals do not recognize deviations in melodic structure (Braun et al., 2008). They demonstrate poor anomalous pitch detection in standardized melodies on such tasks as the Distorted Tunes Test (DTT; Drayna et al., 2001; Braun et al., 2008) and Montreal Battery of Evaluation of Amusia (MBEA; Ayotte et al., 2002; Peretz et al., 2003)^1^. In addition, some individuals with amusia also exhibit deficits in rhythm or beat perception while others are unimpaired on these types of tasks (e.g., Ayotte et al., 2002; Peretz et al., 2003). Individuals with amusia are frequently impaired in vocal production tasks such as singing, though there are exceptions (e.g., Ayotte et al., 2002; Dalla Bella et al., 2009; Tremblay-Champoux et al., 2010), and individuals can be poor singers without having perceptual deficits (Dalla Bella et al., 2007). Furthermore, a dissociation in perception and production skill has been noted in amusia, whereby amusics can sing/hum the correct direction (though not exact interval) of a pitch change in the absence of consciously perceiving it (Loui et al., 2008). Finally, compared to others with typical music perception abilities, amusics generally report less engagement and emotional responsiveness to music compared to individuals without amusia (McDonald and Stewart, 2008).

Research in our laboratory suggest that while musical skill appears to be broadly distributed in WS (Lense and Dykens, 2012), individuals at the bottom of the distribution display marked impairments, similar to those described for TD individuals with amusia. Research is growing on amusia in the general population, including interrelationships among auditory perception, musical production, and musical engagement and emotionality. Amusia thus provides a novel framework for understanding individual differences in musicality in WS.

The concept of amusia occurring in WS may not seem surprising when considering the neural underpinnings of these two disorders. TD individuals with amusia are reported to have decreased white matter in the frontotemporal tracts connecting the right inferior frontal gyrus (IFG) and the right auditory cortex (Loui et al., 2009), as well as decreased white matter in the right IFG itself (Hyde et al., 2006). Electrophysiology and neuroimaging studies suggest that the auditory cortex in TD individuals with amusia responds appropriately to pitch stimuli, while activity in the IFG is decreased compared to individuals without amusia (Peretz et al., 2009; Hyde et al., 2011; Moreau et al., 2013). Thus, amusia appears to be a disorder of disconnectivity rather than strictly auditory perception. Intriguingly, WS is associated with marked reductions in overall white matter, including in the frontal lobe (Reiss et al., 2000; Thompson et al., 2005), as well as abnormal directionality of white matter tracts (Marenco et al., 2007). Thus, despite their auditory sensitivities – and relatively preserved auditory cortices (Reiss et al., 2004; Martens et al., 2010) – rates of amusia may actually be higher in individuals with WS than in the TD population.

The present study is the first to examine how the auditory sensitivities (Levitin et al., 2005) and love of music (Levitin et al., 2004) that characterize WS relate to their variable musical perception and production abilities. First, the percentage of people with amusia was established in a large sample of individuals with WS. Second, in order to understand factors that predict musical pitch perception abilities in WS, relationships were examined among musical pitch perception and cognitive factors, basic auditory sensitivities, auditory processing style (i.e., spectral vs. fundamental processing), musical training, musical engagement, and family musical engagement. Third, contributing factors to individual differences in musical skill in WS were assessed, including demographic and cognitive factors, auditory sensitivity and perception, and musical experiences and environment. Fourth, different levels of auditory perception (basic auditory sensitivities vs. musical pitch perception abilities) were examined as predictors of the musical interest and emotional responsiveness to music that are so common in WS.

## MATERIALS AND METHODS

### PARTICIPANTS

The initial sample included 78 participants with WS recruited from a residential summer camp (*n* = 35) or national WS conference (*n* = 43)^2^. While both the camp and conference recruitment materials advertised musical activities, musical training and interest were not prerequisites to be included in the study. Four conference participants were excluded from analysis because they were unable to understand task directions (*n* = 2) or attend during the testing session (*n* = 2). One camp participant was excluded because his parents reported 40% bilateral hearing loss. Thus, the final sample for the perceptual tasks included 73 participants. One task, a singing exercise, was added to the test battery after data collection had already begun; 60 of the 73 participants had singing data. **Table 1** summarizes demographic data for the final sample.

**Table 1:** Demographic information (*n* = 73).

	Mean ± SD (range)
Age (years)	26.2 ± 9.4 (10–51)
Gender (% male)	49.3
Full Scale IQ	70 ± 14.5 (43–97)
Verbal IQ	76.8 ± 12.0 (54–108)
Non-verbal IQ	69.8 ± 17.1 (40–97)

This study was approved by the Institutional Review Board (IRB) of the university. Parents/guardians of all individuals with WS provided informed consent. All individuals with WS provided verbal assent (and written assent if they were able to do so) after a research assistant read them a short, IRB-approved script explaining the study procedures.

### MEASURES AND PROCEDURE

#### Parent questionnaires

Parents of the WS participants completed several questionnaires about their child:

#### Demographics questionnaire

Parents provided background information about the participant’s diagnosis, hearing loss, history of ear infections, age, gender, family income, and education.

#### Musical questionnaires

The Musical Background Questionnaire (Lense and Dykens, 2012) recorded information about each participant’s type and duration of previous and current formal and informal musical activities, including participation in lessons or ensembles, playing and listening to music, composition, and note reading. Musical training was quantified in two ways. Exposure to training reflected the number of types of formal music lesson experiences (including individual and group lessons outside of or through school, as well as ensemble participation), while duration of individual training was computed as the cumulative duration of participation in individual extra-curricular music lessons. Musical engagement was measured as the number of hours per day currently spent singing/playing an instrument and the number of hours per day currently spent listening to music. The Family Music Background Questionnaire (created for this study) recorded information about the musical activities and training of an individual’s nuclear family. The number of family members who played a musical instrument/sang (at any point in their life) was used to index the family musical environment. On the Music Interest Scale (MIS: Blomberg et al., 1996), parents rated their child on 14 statements about musicality using a 6-point scale, which indexed three subscales: Musical Skill (e.g., “My child has a good sense of rhythm”), Musical Interest (e.g., “My child is always listening to music”), and Emotional Reaction to Music (e.g., “Music makes my child happy”). As musical skills were separately assessed, analyses used the MIS Musical Interest and Emotional Reaction to Music subscales.

#### Sensitivity to sounds

(Lense and Dykens, 2012). Parents rated how bothered or frightened their child is to 21 specific non-musical sounds (e.g., truck engine, clock ticking, helicopter, thunder, etc.) and five different sound characteristics (loudness, suddenness, duration, low pitched, high pitched) on a seven point Likert scale. Total scores reflecting sensitivity to specific (non-musical) sounds and sensitivity to sound characteristics were computed by summing across the 21 specific sounds and five sound characteristics, respectively. These scores were used as indicators of general auditory sensitivities, which were not specific to music.

### BEHAVIORAL ASSESSMENTS

#### Cognitive assessment

Participants were individually assessed with the Kaufman Brief Intelligence Test, 2nd edition (KBIT-2; Kaufman and Kaufman, 2004), which yields verbal, non-verbal, and full-scale IQ scores. This test has been previously used with good success in WS (e.g., Dykens, 2003; Lense and Dykens, 2012; Mervis et al., 2012). The full-scale IQ score was used as an indicator of cognitive abilities.

#### Spectral-fundamental processing

The sound perception test created and utilized by Wengenroth et al. (2010) was administered individually to all participants. This 12-item test is a short 2-minute version of a more extensive psychoacoustic test (Schneider et al., 2005), which assesses an individual’s dominant processing style. Participants heard a pair of tones repeated twice. The tones were 500-ms in duration with 10-ms rise and fall time and 250-ms ISI between tones. The harmonics of the tones varied in number (2, 3, 4), height (low or high partial tones compared to the fundamental frequency), and averaged frequency of harmonics (low = 0.8 kHz, high = 1.5 kHz). Participants reported if the second tone in the pair was higher or lower than the first through verbal report, gesturing the direction, or singing the tones back to the researcher (following the methods of Wengenroth et al. (2010).^3^ Prior to the actual test, participants completed several practice items to ensure that they understood and used the higher/lower terminology appropriately. For each tone pair, the direction of the pitch change perceived by the participant reflected either spectral or fundamental sound perception. An SFP index was computed by the formula (number of spectral perception items – number of fundamental perception items)/total number of items. Scores greater than 0 reflected more dominant spectral perception while scores less than 0 reflected more dominant fundamental (i.e., fundamental frequency) perception. Test-retest reliability for seven individuals with WS has been reported as *r* = 0.95, *p* < 0.001 (Wengenroth et al., 2010).

#### Distorted tunes test

The DTT (Drayna et al., 2001) was used to test pitch amusia. While the DTT has been formally labeled a test of “tune deafness” rather than amusia (Braun et al., 2008), it assesses anomalous pitch detection in otherwise appropriate melodies, a task that has been proposed as a diagnostic marker of amusia (Ayotte et al., 2002)^4^. Participants were presented with 26 well known tunes, nine of which were played correctly and 17 of which were altered to have note errors (Drayna et al., 2001). Tunes ranged from 12 to 26 notes in length (4–14 s). In the altered tunes, the pitches of two to nine notes were changed (usually within 1–2 semitones of the original note) but the rhythm and contour of the melody were unaltered. Following each tune, participants indicated if the tune was played correctly or incorrectly by verbalizing their response or pointing to a happy or sad face, respectively. Participants then reported whether or not they were familiar with the tune though were not required to name the song. To ensure comprehension, participants completed a practice item prior to the test proper. Scores can be examined continuously or dichotomously, with scores ≤18 considered amusic (i.e., below the 10th percentile in TD samples; Jones et al., 2009). Test-retest reliability across a one-year delay for five participants with WS was excellent based on Fleiss (1986) guidelines (ICC = 0.876).

Both perception tasks were presented at 68 dB from two speakers approximately 40 cm in front of the participant.

#### Singing

To examine musical vocal production abilities, participants were shown a picture of a baby having a birthday party and asked to sing “Happy Birthday” to the baby. Performances were recorded directly onto an Apple laptop using a handheld microphone at a sampling frequency of 44.1 kHz. First, participants were recorded singing their own rendition of “Happy Birthday” without any musical prompts (“original rendition”). They were then presented with a recording of a female singer performing “Happy Birthday” with metronome clicks at 88 bpm. Participants sang along with the recording until they felt comfortable singing at that speed. They were then recorded singing “Happy Birthday” at the prescribed tempo (“metronome rendition”).

In order to examine how the singing abilities of individuals with WS are perceived by others, two judges (semi-professional musicians/music educators) independently rated offline the participants’ original renditions of “Happy Birthday.” Judges rated the participants’ vocal tone, intonation, technique, diction, note accuracy, rhythm accuracy, tempo, and interpretation according to a grading rubric that assigned scores on a 0–4 scale. Scores were summed and converted to a percentage for a total score. This rating method has been successfully used with WS participants previously (Lense and Dykens, 2012). For the current study, average measures intra-class correlation for the total score was 0.905 for the two judges.

To examine objective measures of singing abilities in WS, psychoacoustic analyses were conducted on the vowel groups for both their original and metronome renditions using Praat software. Vowel groups were first determined using the Prosogram automatic segmentation tool (Mertens, 2004), which is ~80% precise for singing samples (Salselas and Herrera, 2011). Vowel groups were then manually reviewed to adjust vowel boundaries when the automatic detection erroneously omitted or inserted vowels, typically as a result of very unsteady singing or a poor articulation of phrases. Such automatic and semiautomatic approaches to singing data have been used previously (Tremblay-Champoux et al., 2010; Salselas and Herrera, 2011). Fundamental frequency (F_0_) of each vowel group (in Hz) was computed using the Praat autocorrelation method. Data was manually inspected for false pitch detections (i.e., if the software picked a higher octave than F_0_) and corrected. Note onset times based on vowel onset times were computed in ms. Melodic and temporal variables were computed based on previous studies (e.g., Dalla Bella et al., 2007; Martinez-Castilla and Sotillo, 2008). In the melodic domain, *interval deviation* was computed as the mean absolute difference in semitones between the sung interval and the interval indicated in the score. Lower interval deviation indicates higher relative pitch accuracy. *Percentage of contour errors* was calculated as the percent of sung intervals that differed from the pitch direction change indicated in the score. Thus, the contour errors identified whether or not participants were producing the correct gestalt of the song even if they were unable to sing precise intervals. In the temporal domain, *tempo* was computed based on the mean inter-onset-interval (IOI) of the quarter notes. *Temporal variability* was computed based on the coefficient of variation (CV) of the quarter note IOIs. Both of these variables were considered important because some individuals’ singing improves when they slow down their singing speed (Dalla Bella et al., 2007). Psychoacoustic analyses were unable to be conducted on 5 original and 3 metronome renditions due to highly original singing (i.e., participants did not sing the canonical “Happy Birthday” melody).

### ANALYSES

As this is the first study of amusia in WS, analyses were conducted both continuously, to examine relationships among perception, production, and musical engagement, as well as dichotomously, to examine categorical differences between individuals with WS who do and do not meet criteria for amusia. Unless otherwise stated, non-parametric statistics were used (i.e., Spearman correlations for continuous analyses; Mann–Whitney *U* tests for dichotomous analyses). These approaches were optimal as most participants were not expected to meet criteria for amusia, and, consistent with the general population, the distribution of scores on the DTT was negatively skewed. 95% confidence intervals were constructed using non-parametric bootstrapping methods based on 1000 bootstrapped samples. Linear regressions were conducted to predict unique contributions of the independent variables to musical perception, skill, and emotion. For all regression analyses, residuals were normally distributed, such that parametric linear regressions were considered appropriate.

Preliminary analyses assessed a possible confound related to hearing loss. Individuals with parent-reported hearing loss (6.1%) or history of ear infections (17.2%) were no more likely to meet criteria for amusia than individuals without these hearing issues (χ^2^ < 1.0, *p* > 0.4). Therefore, hearing loss or chronic ear infections were not included as covariates in the analyses.

An exact binomial test was used to compare the rate of amusia in WS versus the TD population (4%; Sloboda et al., 2005). Next, correlations were conducted among scores on the DTT and the SFP tests, age, IQ, musical training (number of types of formal musical lessons; cumulative duration of extra-curricular individual lessons), musical engagement (time currently spent playing music and listening to music), and family musical environment. Variables that were significantly associated with DTT scores were entered into a linear regression analysis to assess their unique contributions to variance in DTT performance.

To examine the relationship between musical perception and production, performance on the DTT was correlated with subjective ratings of singing abilities and the psychoacoustic measures for both original and metronome renditions. Psychoacoustic variables (interval deviation, percent of contour errors, tempo, and tempo variability) were also compared between participants’ original and metronome renditions to assess if singing skill improved following practice with a recording. Correlations were conducted between subjective singing ratings and age, IQ, SFP index, sound sensitivities, DTT, musical training, musical engagement, and family musical environment. Variables that were significantly associated with singing ratings were entered into a linear regression analysis to assess their unique contributions to variance in singing skill.

Last, correlations were conducted for the Musical Interest and Emotional Reaction to Music subscales with DTT score, total sensitivity to specific sounds score, and total sensitivity to sound characteristics score. A linear regression analysis further examined the unique contributions of musical pitch perception abilities (DTT score) and sound characteristic sensitivity to participants’ Emotional Reaction to Music scores.

## RESULTS

### RATES OF AMUSIA

**FIGURE 1:**
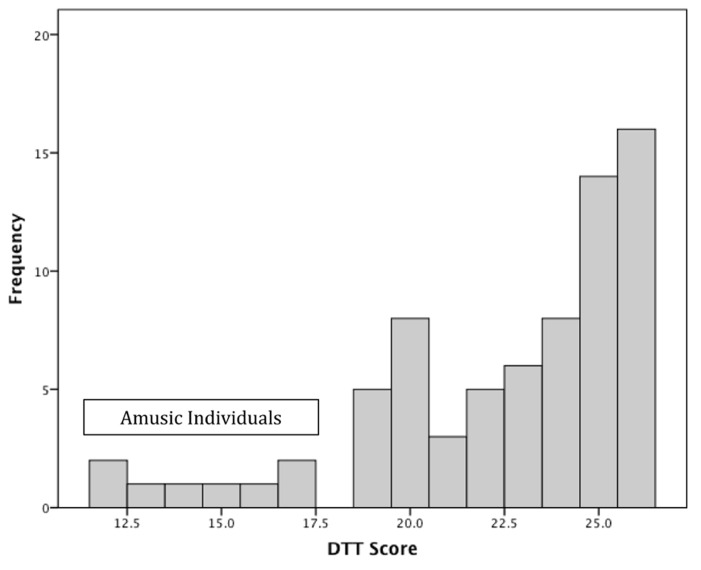
**Distribution of scores on the distorted tunes test (DTT).** Scores ≤18 are considered amusic.

**Figure 1** displays the distribution of scores on the DTT in our WS sample. There was much variability in performance on the DTT, with scores ranging from chance levels to perfect scores, with a mean performance of 22.5 ± 3.7 correct answers. As evident in the figure, no individual scored at the cutpoint of 18, leading to a dichotomous distribution. Of the 73 participants with WS, eight individuals (11%; 95% confidence interval: 4.1–18.6%) had scores ≤18 on the DTT, i.e., in the range of amusia. This occurrence rate of 11% in our sample is significantly greater than the 4% prevalence rate in the TD population based on an exact binomial test (*p* = 0.009).

Response style was examined to better understand differences in performance among individuals who did versus did not meet criteria for amusia. Specifically, hits were defined as the number of correctly played tunes detected as correctly played while false alarms were defined as the number of incorrectly played tune that were responded to as correctly played. Participants with and without amusia did not differ in number of hits (M ± SD: 7.8 ± 1.8 vs. 8.4 ± 1.0, *U* = 228.0, p = NS), but those participants with amusia had more false alarms than their counterparts without amusia (10.3 ± 2.6 vs. 2.0 ± 2.1, *U* = 3.0, *p* < 0.001). Among participants without amusia, false alarms were not related to the proportion of distorted notes in the tune (*r* = 0.232, p = NS) but were more likely to occur when the error notes occurred proportionally earlier in the tune (proportional placement of first error note: *r* = –0.508, *p* = 0.037; proportional placement of last error note: *r* = –0.34, *p* = 0.082). In contrast, there was a trend for participants with amusia to make more false alarms when there were proportionally more error notes in the tune (*r* = 0.474, *p* = 0.055). There were no associations between false alarms and the placement of the error notes for participants with amusia (*p*’s > 0.2). Thus, as error notes did not systematically shift the tonality of the tune, participants with amusia made more false alarms even with increasing numbers of cues to the distorted nature of the tune.

### MUSICAL PITCH PERCEPTION, AGE, IQ, SOUND SENSITIVITY, AUDITORY PROCESSING, AND MUSICAL ENGAGEMENT VARIABLES

**FIGURE 2:**
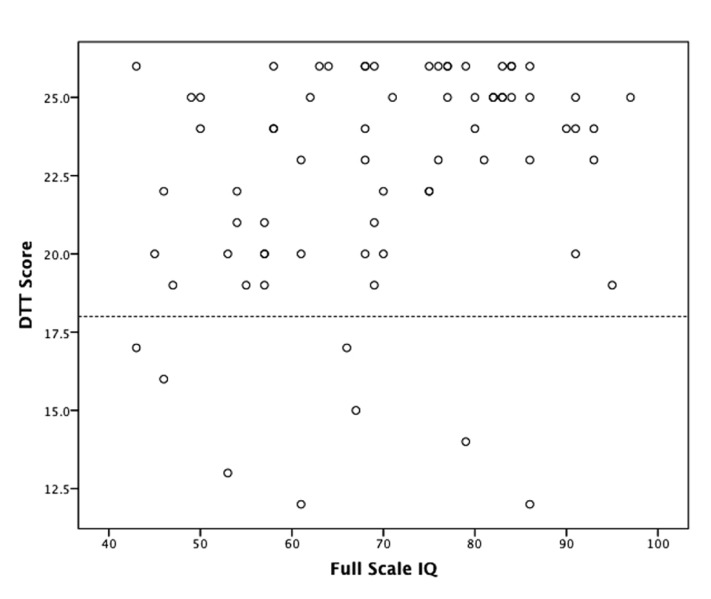
**Relationship between distorted tunes test (DTT) and IQ.** Scores below the dashed line are in the range of amusia (≤18). There was a moderate association between DTT and IQ (ρ = 0.306, *p* = 0.008).

There was no association between DTT performance and age (ρ = 0.069, p = NS) but there was a moderate association between DTT and IQ (ρ = 0.306, *p* = 0.008). IQ scores showed similar ranges in those with (range 43–86) versus without amusia (range 43–97) and mean IQ scores did not differ across these groups (see **Figure 2**). Subsequent correlations were conducted with and without controlling for IQ; effects did not differ, so only the zero-order correlations are presented below.

Distorted tunes test performance was not related to sensitivity to specific (non-musical) sounds (ρ = -0.167, p = NS) or sensitivity to sound characteristics (ρ = -0.084, p = NS), suggesting that amusicality was not due to differences in general auditory sensitivities. As well, no significant association emerged between DTT scores and family music environment (ρ = -0.012, p = NS), indicating that amusicality does not appear to be due to an impoverished family musical environment.

Over 90% of participants had received some type of musical training, which most commonly involved extra-curricular private music lessons (64.8%) and/or participation in such ensemble activities as the school band, rock band, or church choir (80.3%). Participants were typically trained on piano (35.2%), drums/percussion (25.4%), and voice (23.9%), though a variety of other instruments were represented (e.g., violin, guitar, saxophone). Information on the musical training of the study participants is presented in **Table 2**. There were moderate associations between DTT score and musical training (number of types of music lessons: ρ = 0.461, *p* < 0.001; cumulative duration of individual musical training: ρ = 0.330, *p* = 0.005) but no relationship between DTT and current time spent playing music (ρ = 0.153, p = NS) or current time spent listening to music (ρ = -0.085, p = NS).

**Table 2:** Musical background and skill.

	Mean ± SD
Types of musical training	2.8 ± 1.9
Cumulative duration of individual extra-curricular music lessons (yrs)	5.7 ± 8.6
Time currently play music (hrs)	1.2 ± 1.9
Time currently listen to music (hrs)	3.1 ± 2.5
MIS – Musical Interest	17.9 ± 5.2
MIS – Emotional Reaction to Music	15.3 ± 5.2
Sensitivity to specific (non-musical) sounds	50.1 ± 19.1
Sensitivity to sound characteristics	18.2 ± 6.4
Number of family members who play music	2.1 ± 1.4
Rating of singing abilities (%)	42.2 ± 16.4

*MIS, Music Interest Scale*

Scores on the SFP ranged from -1 (extreme fundamental processing preference) to 0.5 (somewhat spectral processing preference), with a mean indexing a somewhat fundamental processing preference (-0.24 ± 0.42). DTT and SFP scores were negatively associated (ρ = -0.276, *p* = 0.018), indicating that individuals who did poorer on the DTT (consistent with amusia) generally performed in the mixed or somewhat spectral range. To further understand this relationship, SFP scores were divided into three categories – mostly fundamental processing (scores ranging from -1 to -0.5), mixed processing (scores greater than -0.5 but less than 0.5), and mostly spectral processing (scores ranging from 0.5 to 1). All eight amusic participants scored in the mixed processing category, while non-amusic participants were split among the fundamental (35.4% of non-amusics), mixed (61.5%) and spectral (3.1%) processing preference categories (Fisher’s exact test *p* = 0.088). Thus, while non-amusic participants varied in their processing preference from extreme fundamental to somewhat spectral processing, all amusiac individuals demonstrated a mix of fundamental and spectral processing.

A regression analysis including variables significantly associated with DTT scores [i.e., IQ, musical training (exposure to training; duration of individual training), SFP] accounted for 23.4% (adjusted *R*^2^) of the variance in DTT performance (*F*_4,66_ = 6.353, *p* < 0.001; see **Table 3**). However, only exposure to music training (i.e., number of types of musical lessons, β = 0.378, *t* = 2.518, *p* = 0.014, s*r*^2^ = 0.069) was a significant predictor of DTT performance after controlling for other significant bivariate correlates of DTT.

**Table 3:** Regression model predicting performance on the distorted tunes test.

Predictor	β	*t*	*p*	Semipartial *r*
(Constant)		8.633	<0.001	
IQ	0.198	1.841	0.070	0.193
SFP index	-0.143	-1.314	0.193	-0.137
*Exposure to musical training*	*0.378*	*2.518*	*0.014*	*0.263*
Duration of individual music training	0.028	0.197	0.844	0.021

*SFP index, spectral fundamental processing index. Significant predictors are italicized.*

### MUSICAL PITCH PERCEPTION AND SINGING SKILL

**FIGURE 3:**
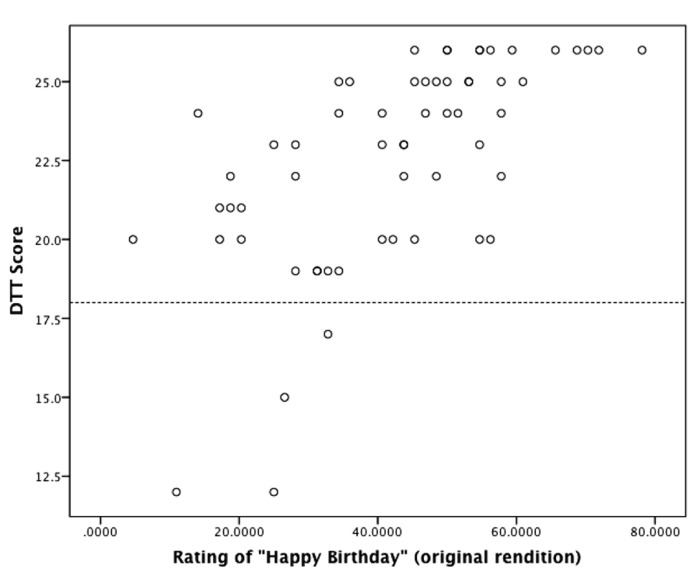
**Relationship between performance on the distorted tunes test (DTT) and ratings of singing abilities.** Scores below the dashed line are in the range of amusia (≤18). There was a strong association between musical perception abilities (DTT) and production abilities (singing; ρ = 0.684, *p* < 0.001).

Ratings of participants’ singing abilities were normally distributed and reflected a wide range of musical skill (42.2% ± 16.4%, range 4.6–78.1%). Participants’ DTT scores were associated with ratings of their singing abilities (ρ = 0.684, *p* < 0.001; see **Figure 3**). Consistent with this, psychoacoustic analyses demonstrated that greater performance on the DTT was associated with less interval deviation (ρ = -0.601, *p* < 0.001) and fewer contour errors (ρ = -0.509, *p* < 0.001). DTT score correlated with tempo (ρ = 0.340, *p* = 0.011) indicating that individuals who performed better on the DTT sang at a slower tempo. However, controlling for tempo did not change the relationships between DTT and interval deviation (ρ = -0.544, *p* < 0.001) or contour errors (ρ = -0.466, *p* < 0.001). Additionally, temporal variability (i.e., the stability of the tempo of their singing) did not correlate with DTT performance (ρ = -0.034, p = NS; controlling for tempo: ρ = -0.062, p = NS).

Compared to their own original renditions of “Happy Birthday,” their singing after practice with a recording and metronome was significantly slower (quarter note IOI: 0.515 ± 0.119 ms vs. 0.608 ± 0.088 ms, *t*_53_ = -6.085, *p* < 0.001) with less interval deviation (0.98 ± 0.57 vs. 0.86 ± 0.46 semitones, *t*_53_ = 2.237, *p* = 0.029). The decreases in percentage of contour errors (7.2% ± 5.8% vs. 6.2% ± 5.1%) and temporal variability (0.238 ± 0.117 vs. 0.222 ± 0.093) were not significant. All associations between DTT score and singing of their original renditions were also found between DTT score and singing of their metronome renditions (interval deviation: ρ = -0.613, *p* < 0.001; percentage of contour errors: ρ = -0.466, *p* < 0.001; tempo: ρ = 0.310, *p* = 0.019), while temporal variability continued not to be associated with DTT performance (ρ = -0.126, p = NS; the same results were found when controlling for tempo).

To examine factors that contribute to singing abilities, ratings of singing abilities were correlated with age, IQ, sound sensitivities, auditory processing style (SFP index), number of family members who play/sing, DTT score, musical training, and current time spent playing and listening to music. The six significant variables (IQ: ρ = 0.255, *p* = 0.05; SFP index: ρ = -0.282, *p* = 0.029; DTT score: ρ = 0.684, *p* < 0.001; music lesson exposure: ρ = 0.407, *p* = 0.002; duration of individual training: ρ = 0.369, *p* = 0.004; current time playing music: ρ = 0.339, *p* = 0.01) were then entered into a regression analysis, which accounted for 59.9% (adjusted *R*^2^) of the variance in ratings of singing abilities (*F*_6,50_ = 14.932, *p* < 0.001, see **Table 4**). However, only three variables uniquely contributed to singing abilities: DTT score (β = 0.546, *t* = 5.678, *p* < 0.001, s*r*^2^ = 0.231), duration of individual training (β = 0.344, *t* = 3.091, *p* = 0.003, s*r*^2^ = 0.069), and current time spent playing music (β = 0.341, *t* = 3.572, *p* = 0.001, s*r*^2^ = 0.091). IQ, auditory processing style, and exposure to musical training did not predict singing abilities.

**Table 4:** Regression model predicting singing abilities.

Predictor	β	*t*	*p*	Semipartial *r*
(Constant)		-3.152	0.003	
IQ	0.180	1.863	0.068	0.158
SFP index	-0.129	-1.407	0.166	-0.119
*DTT*	*0.546*	*5.678*	*<0.001*	*0.481*
*Current time play music*	*0.341*	*3.572*	*0.001*	*0.302*
Exposure to musical training	-0.080	-0.670	0.506	-0.057
*Duration of individual music training*	*0.344*	*3.091*	*0.003*	*0.262*

*SFP index, spectral fundamental processing index; DTT, distorted tunes test. Significant predictors are italicized.*

### MUSICAL PITCH PERCEPTION, SOUND SENSITIVITIES, MUSICAL INTEREST, AND EMOTIONAL REACTIONS TO MUSIC

Musical interest was not associated with sensitivity to sound characteristics (ρ = 0.112, p = NS) or sensitivity to specific (non-musical) sounds (ρ = -0.162, p = NS), but was moderately associated with DTT performance (ρ = 0.329, *p* = 0.005). Emotional reaction to music was moderately associated with sensitivity to sound characteristics (ρ = 0.312, *p* = 0.008) but not to sensitivity to specific (non-musical) sounds (ρ = -0.007, p = NS). There was a small-to-moderate association between emotional reaction to music and DTT score (ρ = 0.235, *p* = 0.047).**

A regression analysis was conducted to further examine the relationship between auditory sensitivities and emotional reaction to music because low-level auditory sensitivities and/or sensitivity to melodic structure represent two possible pathways underlying the emotional responsiveness to music in WS. Sensitivity to sound characteristics and DTT scores together explained 9.5% (adjusted *R*^2^) of the variance in emotional reaction to music. However, sensitivity to sound characteristics (e.g., the sound volume, duration, articulation, frequency) entirely accounted for this relationship (β = 0.318, *t* = 2.782, *p* = 0.007, s*r*^2^ = 0.100). DTT scores, which reflect sensitivity to melodic structure, had no significant predictive value (β = 0.174, *t* = 1.527, *p* = 0.131, s*r*^2^ = 0.030).

### DICHOTOMOUS ANALYSES

Dichotomous analyses were conducted to mirror approaches commonly used in the TD literature of individuals with versus without amusia. The 8 individuals with amusia had fewer types of musical training than individuals without amusia (1.3 ± 1.0 vs. 3.0 ± 1.9 lesson types, *U* = 114.5, *p* = 0.011) although they did not significantly differ in their cumulative duration of individual training (3.6 ± 7.5 vs. 6.0 ± 8.7 years, *U* = 182.5, p = NS). Individuals with and without amusia also did not differ on IQ, SFP index, sensitivity to specific (non-musical) sounds, sensitivity to sound characteristics, time currently spent playing or listening to music, or family musical environment (*U*’s > 173, *p*’s = NS). Of the individuals with singing data, four met criteria for amusia. These individuals had poorer singing abilities based on subjective ratings, as well as the psychoacoustic measures of interval deviation in both renditions and the percentage of contour errors in the metronome rendition (*U*’s < = 33, *p* < .03).

## DISCUSSION

This study is the first to examine individual differences in musicality in WS through the lens of amusia, and the largest to date to use direct behavioral testing of musical perception and skill in this genetic disorder. In doing so, we made a conceptual shift away from previous work emphasizing musical talent, relative sparing or strengths, and instead placed musicality in WS in a framework that reflects current music cognition research. Results thus have important implications for our understanding of the complex auditory phenotype in WS. Furthermore, results demonstrate how a genetic, neurodevelopmental disorder can uniquely inform our understandings of genetic and musical training contributions to musicality in both typical and atypical populations.

We found a distribution of scores on the DTT in our sample of 73 adolescents and adults with WS that was remarkably similar to distributions from large TD samples, with scores ranging from chance levels to perfect performance (Drayna et al., 2001; Jones et al., 2009). Amusia aside, it is worth noting that 52% of our WS sample had DTT scores in the 24–26 points range, as did 58% of 864 individuals in a TD sample (Jones et al., 2009), consistent with scoring above the 60th percentile in TD distributions. Similarly, 22% of our WS sample demonstrated perfect performance on the DTT, consistent with the 26% of TD individuals reported in Jones et al. (2009). Thus, many individuals with WS performed quite well on the DTT at rates similar to those reported in TD samples.

Even so, 11% of our sample of 73 individuals with WS met criteria for amusia; a higher rate than the 4% rate reported in the TD population. Intriguingly, however, our 11% rate is remarkably similar to other studies using targeted TD groups that were biased toward higher rates of amusia. Specifically, Cuddy et al. (2005) reported that 11% of 100 undergraduate students who self-reported as tone deaf actually met the criteria for amusia using the MBEA. Also, in a family aggregation study, 11% of 37 offspring of amusic individuals met criteria for amusia using the MBEA (Peretz et al., 2007). Finally, we found a dichotomous distribution for individuals with WS who did versus did not meet criteria for amusia, a pattern also reported for tasks similar to the DTT (but using different melodies) in TD samples (Ayotte et al., 2002). Thus, the frequency of amusia in our WS sample was higher than in the general TD population but similar to studies of targeted TD groups at higher risk for amusia. Taken together, findings suggest that amusia is a useful framework for better understanding musicality in WS, and that future population-based studies are needed to ascertain true prevalence rates of amusia in WS.

Though DTT scores were moderately associated with IQ, IQ scores in both the amusic and non-amusic groups were variable and included individuals who ranged from moderate levels of intellectual disability to those in the average range of cognitive function. Thus, factors aside from IQ are important for DTT performance. As previous studies have typically only included individuals with average intellectual functioning, our study indicates that amusia can also be successfully examined in people with cognitive limitations.

Individuals with versus without amusia differed in response style – amusics had more false alarms than those without amusia and increased numbers of error cues in the tunes did not reduce their false alarm rate. This response style – reporting that incorrect melodies were played correctly – is consistent with several previous studies of amusia in general. On a pitch memory task, amusics were more likely to incorrectly report that the differing final and initial pitches in a sequence of tones were the same (Williamson et al., 2010). Amusics also frequently report that melodies with mistuned or out-of-key notes sound correct (Peretz et al., 2009). Additionally, in a melodic same/difference task from the MBEA, amusics were more likely to respond that melodies with pitch deviations were the same as the original melody using signal detection analyses (Henry and McAuley, 2013). This bias for perceiving melodies as in tune or the same is consistent with EEG studies indicating lack of conscious awareness of pitch deviations in amusia (Peretz et al., 2009; Moreau et al., 2013). Future WS studies could build on this finding by using EEG or other imaging methods to examine neural correlates of pitch perception (or lack thereof).

Our results also suggest that musical perception in WS is not related to general auditory sensitivities, based on the near-zero correlations with the measures of sound sensitivity to non-musical sounds and sensitivity to sound characteristics. Similarly, individuals with amusia in the general population can recognize environmental sounds without difficulty (Ayotte et al., 2002; Marin et al., 2012). Additionally, TD individuals with amusia appear to have intact basic discrimination of musical timbres (Marin et al., 2012). Though not addressed in the current study, previous work finds intact musical timbre discrimination in WS (Lense et al., 2012).

Implications of the SFP findings are less clear-cut. Though amusia was generally associated with a more mixed versus fundamental auditory processing style, many individuals without amusia also demonstrated mixed auditory processing. Furthermore, processing style did not uniquely predict DTT performance in the regression analysis. Though our sample overall demonstrated a tendency toward fundamental processing, this preference was not nearly as strong as in a previous study (Wengenroth et al., 2010). Differences in sample size, age, response style, and musical training and experiences may contribute to these somewhat discrepant findings. For example, WS participants in the current study tended to respond verbally (i.e., explicitly) to the SFP stimuli, while the majority of participants with WS in the prior study automatically responded by singing (i.e., implicitly (though their TD controls preferred to respond verbally); M. Wengenroth, personal communication, July 3, 2013). As Loui et al. (2008) reported discrepant explicit vs. implicit responses to pure tone pairs in amusics (but not non-amusics), future studies should examine whether response style affects perception of pure tone, complex tone, and SFP-type stimuli (complex tones missing the fundamental frequency) in amusic and non-amusic participants with WS.

No relationships emerged between family musical environment and DTT performance, suggesting that amusic individuals with WS are not simply from non-musical families. In contrast, a family aggregation study indicated that TD individuals with amusia had fewer family members who were amateur musicians than did individuals without amusia, though all had at least one sibling who did not have amusia (Peretz et al., 2007). Amusia has a high heritability (Drayna et al., 2001; Peretz et al., 2007), and future research will need to directly examine musical perception and production abilities in WS family members. In this vein, WS is an ideal model system for sorting out unique versus shared contributions of genetic, neural, family environmental and music training factors in the development of musical skill.

Indeed, exposure to musical training was the strongest predictor of DTT performance, accounting for ~7% of the variance in DTT scores. Behavioral genetics studies indicate that environmental variables, including musical experiences, account for 20–29% of DTT variance (Drayna et al., 2001). In some regards, our findings of amusia in WS are striking when considering the ubiquity of musical engagement in our sample: Over 90% of our sample (and 75% of amusic participants) had at least one type of musical training and nearly two-thirds received individualized private instruction. This pattern of musical perception impairment despite some type of musical training is consistent with descriptions of TD individuals with amusia (e.g., Ayotte et al., 2002; Peretz and Hyde, 2003). Though it may seem surprising that exposure to types of musical training and not duration of individual lessons predicted DTT scores, this most likely reflects both the psychometrics of the DTT (most participants do quite well regardless of training exposure) and the diversity of musical training experiences available to participants with WS. Some parents, for example, reported that they were unable to initiate or maintain extra-curricular music lessons for their child because music teachers were unable or unwilling to work with children with disabilities in their area. Others reported that early attempts to teach their child an instrument sometimes failed because of fine motor demands, which discouraged them from continuing independent lessons. However, these same individuals might successfully participate in an ensemble through their school or religious institution during adolescence. These findings suggest that researchers may need to assess a variety of musical training experiences, not just independent lessons, to best understand musicality in populations with disabilities. This relationship between musical perception abilities and training will be further discussed below in the context of musical skill.

Consistent with previous studies (Ayotte et al., 2002; Dalla Bella et al., 2009; Tremblay-Champoux et al., 2010), poorer performance on the amusia measure was associated with poorer singing abilities, which was not explained by the tempo of their singing. Using psychoacoustic analyses, amusia was particularly associated with poor performance on the pitch dimensions of singing while temporal variability was not associated with DTT score. Greater singing impairment on the pitch versus temporal dimension is commonly seen in amusia, as not all amusics exhibit temporal impairments (Tremblay-Champoux et al., 2010). Interestingly, rhythm abilities have been cast as a relative strength in WS (Levitin and Bellugi, 1998; Lense and Dykens, 2011). The one prior study using psychoacoustic analysis of singing abilities in WS found that their temporal variability did not differ from that of TD participants (Martinez-Castilla and Sotillo, 2008). Thus, it is possible that pitch impairments may be a more common occurrence in WS than temporal impairments. It is also possible that the lyrics guided participants to have consistent temporal variability regardless of their pitch perception abilities. Indeed, some amusics are unable to sing a song without the lyrics to guide them (Dalla Bella et al., 2009; Tremblay-Champoux et al., 2010). Although having participants practice with a recording and metronome slowed down their singing and somewhat improved their interval accuracy, it did not meaningfully alter the relationship between their perceptual and production skills. This consistent impairment in singing on one’s own and imitating another’s singing has also been previously documented in TD amusic individuals (Ayotte et al., 2002).

Intriguingly, in the regression analysis examining predictors of musical skill, DTT performance was the most prominent unique predictor of singing abilities, accounting for 23.1% of the variance in singing skill. Current time spent playing music explained an additional 9.1% of singing variance and cumulative duration of individual extra-curricular lessons explained 6.9% of variance. In contrast, exposure to musical training experiences accounted for less than 1% of the variance in singing ability. DTT and musical training variables were moderately related, yet DTT performance was much more salient in predicting musical production abilities. These relationships suggest that individuals who are less able to pick up melodic structure may be less likely to engage in as much musical training and/or to translate musical training into musical production skills. Lense and Dykens (2012) found that even beyond prior musical skill, endorsement of auditory learning strategies predicted learning of a new instrument in a one-time training session in individuals with WS. Thus, auditory awareness and ability likely interact with musical exposure and training in determining the development of musical skill in WS. Longitudinal and musical intervention studies are needed to better understand musicality throughout development and the effects of specific music training in WS.

In contrast to musical skill, a different pattern of results emerged among auditory perception and musical interest and emotionality. The moderate association between the DTT and the Musical Interest subscale is consistent with reduced engagement with music in everyday life in TD individuals with amusia, although substantial heterogeneity in amusics’ musical enjoyment has also been noted (McDonald and Stewart, 2008; Omigie et al., 2012). Similarly, small-to-moderate associations between the DTT and the Emotional Reaction to Music subscale is in keeping with the finding that people with amusia generally report less psychological change in response to music (McDonald and Stewart, 2008). However, the regression analysis revealed that sensitivity to sound characteristics, and not DTT, predicted emotional responsiveness to music in our WS sample. Therefore, though tonal expectancies are believed to contribute to the emotional experience of music for TD individuals (Steinbeis et al., 2006), for individuals with WS, the physical qualities of music, such as timbre, dynamics and articulation, may be more salient. Low-level or bottom-up processing of these physical features may thus be important for the emotional connection to music in WS. This explanation fits with the general auditory sensitivities in WS (Levitin et al., 2005), and with intense, long-lasting emotional reactions to music experienced by people with WS, even in the face of their poorer musical perception abilities (Don et al., 1999; Levitin et al., 2004). As well, a recent EEG study in WS demonstrated significant differences in alpha band activity, reflective of sensory and attentional processing, within the first 500 ms following the onset of differently valenced emotional music (Lense et al., 2013). Thus, whereas awareness of melodic musical structure was important for musical skill, general auditory sensitivities were more prominent for the emotional connection to music in WS.

Although this study is the first to rigorously examine amusia in a genetic disorder involving cognitive impairments, previous work has examined amusia in relation to language difficulties. For example, poor pitch perception on the DTT was associated with impaired phonemic and phonologic awareness in adults (Jones et al., 2009), and perception-production discrepancies were associated with poor phonemic awareness in children (Loui et al., 2011). Some researchers have also reported pitch-based impairments in children with dyslexia, including poorer abilities to recognize local pitch changes in tone sequences (Ziegler et al., 2012) and impaired detection of incongruous speech pitch (Santos et al., 2007). However, others report intact pitch discrimination abilities in children with dyslexia (Overy et al., 2003). Future studies are needed that directly compare the relationship between pitch perception and phonologic awareness across these and other neurodevelopmental disorders. Additionally, future WS studies are needed that extend beyond IQ by examining relationships among musical, phonologic, and linguistic functioning. Interestingly, musically trained individuals with WS had greater memory for sung than spoken stimuli, whereas individuals without musical training did not show this benefit (Martens et al., 2011). These findings compel future work on relationships among pitch perception, musical engagement, phonological awareness and enhanced musical memory in WS and other neurodevelopmental disorders.

There were several limitations to this study, which point to future research directions. First, rates of hearing loss, which were based on parent report, were lower than expected based on the prevalence of high frequency hearing loss in WS in the literature (Cherniske et al., 2004; Gothelf et al., 2006). The potential of underreported hearing loss may have impacted auditory perception abilities. However, with the exception of one individual who was excluded from analysis, individuals with parent-endorsed hearing loss did not perform in the amusic range on the DTT. Additionally, there was no association between DTT and sound sensitivities, suggesting DTT performance was not due to general auditory functioning. Finally, at least one study matched amusic and non-amusic TD groups on hearing loss (including high frequency hearing loss), suggesting that group differences cannot be entirely explained by potential hearing loss (Ayotte et al., 2002). Nevertheless, future researchers should conduct audiologic testing when examining auditory perception abilities.

Another limitation was that musical production skill was limited to singing, which was done for standardization across participants and comparison with studies of singing abilities in TD amusics (Ayotte et al., 2002; Dalla Bella et al., 2009; Tremblay-Champoux et al., 2010). However, many individuals with WS play instruments such as piano or drums, and their skill on these instruments may be different than their singing abilities. Indeed, the deletion of the elastin gene in WS (Ewart et al., 1993) results in elastin deficiency of the vocal cords (Vaux et al., 2003), which may impede their singing abilities. Incorporating participants’ instruments of choice into future studies may further our understanding of musical perception-production relationships in WS.

Another concern is that sound sensitivities and emotional responsiveness to music were measured via parent report. However, these measures have shown concordance with behavioral and neural markers in other studies (Lense and Dykens, 2012; Lense et al., 2013). Even so, self-report or physiological markers (e.g., skin conductance as a measure of arousal) may further elucidate the role of auditory sensitivities in the emotional connection to music in WS. Future studies are also needed on the role of temporal and rhythmic aspects of music in their emotional responsiveness. Indeed, when amusics do use music to change a psychological state, it is generally along the arousal rather than valence dimension, and with a preference for music with salient rhythms (Omigie et al., 2012). Given the preference for percussion instruments in WS, the temporal qualities of music may also be an important source of emotional responsiveness.

Despite a growing awareness among researchers who work directly with individuals with WS that not all with the syndrome exhibit musical talent, the lore of greater musical skill in WS persists (e.g., Leung, 2009; McPherson and Hallam, 2009; Lovett, 2012). Characterizing WS through this lens of musical talents and strengths may impede our understanding of the vast variability that exists in this syndrome. This study demonstrates the importance of examining individual differences in musicality in WS, with an emphasis on understanding relationships across different levels of auditory perception, production, and emotion. Characterizing the WS auditory phenotype through the lens of amusia rather than preserved skill may prove to be important for ongoing efforts to map gene-brain-behavior relationships; longitudinal studies will be critical for our understanding of how these relationships unfold. Furthermore, amusia in WS may provide a novel window into genetic and neural aspects of amusia in the general population.

## Conflict of Interest Statement

The authors declare that the research was conducted in the absence of any commercial or financial relationships that could be construed as a potential conflict of interest.

## Footnotes

1 Studies employing the DTT typically refer to tune-deafness while studies employing the MBEA utilize the term amusia. This inability to detect anomalous pitches is a hallmark of these amusic/tune-deaf individuals (Drayna et al., 2001; Ayotte et al., 2002) regardless of the terminology. For purposes of this paper, the term “amusia” will be used.

2 Given the low prevalence of amusia in the general population (~4%; Kalmus and Fry, 1980), it is beyond the scope of this paper to include a TD matched control group for comparison purposes. Amusia was defined using the cutoff scores established in the TD literature (Jones et al., 2009). A convenience sample of 22 TD local participants completed the DTT under our testing conditions (described below), with one individual (4.5%) meeting criteria for amusia, consistent with the previous literature.

3 Over 90% of participants in the current study responded verbally; three embellished some of their verbal responses with gestures. The few participants who responded by singing scored in the fundamental processing range on the SFP and the non-amusic range on the DTT.

4 While the MBEA is the most widely used test for amusia in the general population, the DTT was a better fit for the unique characteristics of our WS sample. In particular, the MBEA is a same/difference task involving novel melodies that respondents need to hold in working memory. In contrast, the DTT uses familiar melodies and does not require participants to hold melodies in working memory. Because auditory working memory can be a relative weakness in WS (Don et al., 1999; Rhodes et al., 2011) we were concerned that the MBEA would introduce a study confound and place additional cognitive burdens on our WS participants. Additionally, the familiarity of the distorted tunes on the DTT makes the test very engaging for participants. As several large-scale studies have employed the DTT (e.g., Drayna et al., 2001; Jones et al., 2009) and it tests pitch detection in a manner that has been called “diagnostic” of pitch amusia (Ayotte et al., 2002, p. 250), we felt it was appropriate to use with our WS sample.
